# Transmission comb of a distributed Bragg reflector with two surface dielectric gratings

**DOI:** 10.1038/srep21125

**Published:** 2016-02-19

**Authors:** Xiaobo Zhao, Yongyou Zhang, Qingyun Zhang, Bingsuo Zou, Udo Schwingenschlogl

**Affiliations:** 1 Beijing Key Lab of Nanophotonics & Ultrafine Optoelectronic Systems and School of Physics, Beijing Institute of Technology, Beijing 100081, China; 2 King Abdullah University of Science and Technology (KAUST), Physical Science and Engineering Division (PSE), Thuwal 23955-6900, Saudi Arabia

## Abstract

The transmission behaviour of a distributed Bragg reector (DBR) with surface dielectric gratings on top and bottom is studied. The transmission shows a comb-like spectrum in the DBR band gap, which is explained in the Fano picture. The number density of the transmission peaks increases with increasing number of cells of the DBR, while the ratio of the average full width at half maximum to the corresponding average free spectral range, being only few percent for both transversal electric and magnetic waves, is almost invariant. The transmission peaks can be narrower than 0.1 nm and are fully separated from each other in certain wavebands. We further prove that the transmission combs are robust against randomness in the heights of the DBR layers. Therefore, the proposed structure is a candidate for an ultra-narrow-band multichannel filter or polarizer.

A distributed Bragg reflector (DBR) is an important element widely used in optics^1^,^2^,^3^,^4^,^5^, photonics^6^,^7^,^8^, solar cells^9^, and other fields. It attracts plenty of attention due to its high tunability and extensibility, which can be enhanced by introducing additional structures, for example, defects and gratings. The most important applications of the DBR are optical switches^10^,^11^, lasers^12^,^13^,^14^, sensors^15^, couplers^3^, and narrow-band filters^2^,^4^,^16^. Narrow-band filters can be realized by a semiconductor microcavity that consists of two DBRs and one cavity layer^17^,^18^. When the cavity layer is thick enough, the transmission of the semiconductor microcavity is comb-like with the peak separation being roughly inversely proportional to the thickness, namely, the distance between the two DBRs^4^. In this case, the semiconductor microcavity can serve as a multichannel optical filter. As compared to frequency combs, the comb-like transmission also has substantial advantages in optical frequency metrology^19^, broadband gas sensing^20^, and molecular fingerprinting^21^. Since the transmission peaks are located in the band gap of the DBR, a small peak separation in the comb-like transmission is required. As a result, the cavity layer thickness must be much larger than the photon wavelength. We propose an approach to avoid this requirement. In particular, we achieve a comb-like transmission spectrum in the DBR band gap that is appropriate for optical filters and polarizers with ultra-narrow transmission bands. Also, the transmission combs are robust against randomness in the heights of the DBR layers, which is a key advantage.

## Results

**Figure 1:**
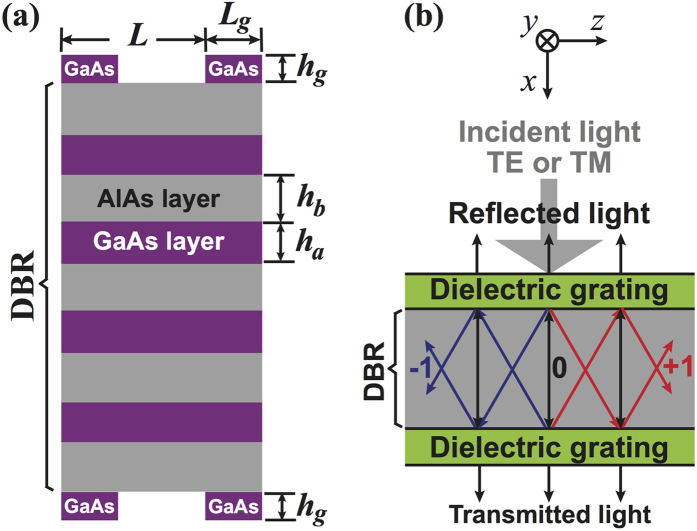
(**a**) Schematic illustration of a DBR with identical GaAs gratings on top and bottom, referred to as G/DBR/G. (**b**) Geometry of the diffraction waves that can exist in the G/DBR/G.

We consider an optical structure consisting of a DBR with two identical dielectric gratings on top and bottom, as shown in Fig. 1(a). We refer to this sandwich structure as G/DBR/G and to the DBR with only one surface dielectric grating as G/DBR. In the G/DBR/G there is no cavity layer similar to that contained in the Fabry-Pérot cavity^18^. The G/DBR/G allows us to achieve a multichannel optical filter, simultaneously with a size reduction. Grating geometries have been applied in many kinds of optical structures to enhance or change the device properties^2^,^15^,^16^,^22^,^23^,^24^. In the present work, the two gratings are introduced to adjust the transmission spectrum in the DBR band gap centered at wavelength 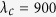 nm. The DBR consists of GaAs and AlAs layers with heights of 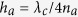 and 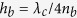 and refractive indices of 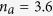 and 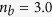
25, respectively. We assume that the DBR has 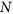 layers of GaAs and 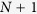 layers of AlAs, totalling to 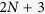 layers for the structure in Fig. 1(a). We also assume that the GaAs gratings on top and bottom coincide in the horizontal direction and have the same height 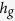, width 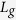, period 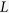, and thus duty cycle 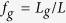. Without loss of generality, the medium above and below the DBR is set to be air with refractive index 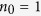. The proposed structure could be fabricated by routine etching of the bottom grating on the substrate, then depositing the DBR, and finally etching the top grating.

The period of the GaAs gratings is set to 300 nm to match the DBR band gap, which implies that the reciprocal lattice vector 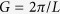 is about 20 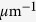. For wavelengths between 860 nm and 940 nm there are only three modes for that 
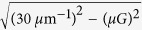
 is real. Only these 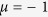, 0, 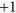 order diffraction waves can exist in the DBR, see Fig. 1(b). Therefore, the electromagnetic wave can be expanded as


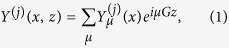


where 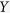 represents the 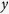 component of the electric field for the transversal electric (TE) mode or magnetic field for the transversal magnetic (TM) mode, see the coordinate system in Fig. 1(b), and the 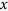 and 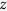-components are zero. For the 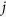
th layer, the coupled equations for 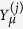 can be written as^2^


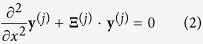


where 
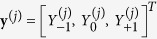
. The matrix 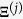 is given by


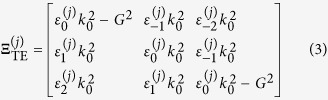


for the TE mode and


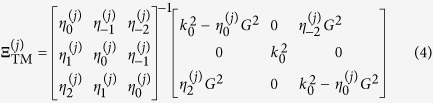


for the TM mode. Here, 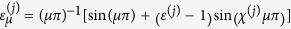 and 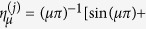

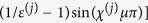
 represents the 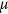
th order Fourier component of the 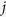
th layer permittivity 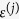 and 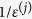, respectively. 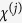 is the duty cycle of the 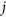
th layer, and equals 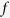 for the grating layers and 1 otherwise. With the help of 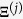, one can find the transfer matrix, 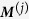, of 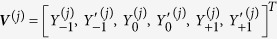 for the 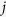
th layer. The derivation is similar to the method developed in ref. 2. Using 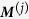, we obtain the total transfer matrix as


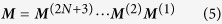


and then the transmissivity 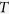 and reflectivity 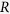.

**Figure 2:**
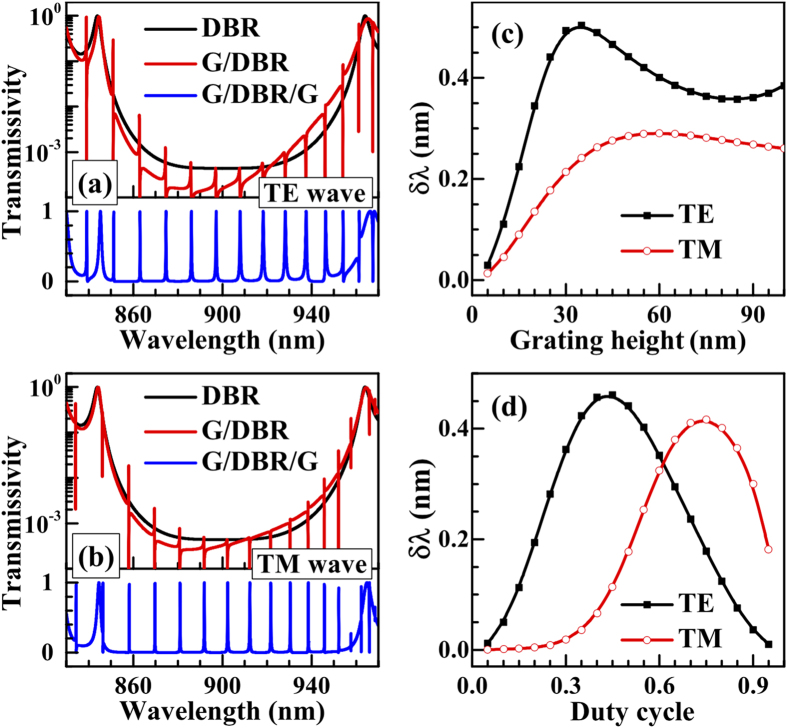
Transmission spectra of the (**a**) TE wave and (**b**) TM wave for the DBR, G/DBR, and G/DBR/G. The vertical axises in (**a**,**b**) are separated into logarithmic and linear regions. In (**c**,**d**) the variation of the average FWHM with the grating height and duty cycle are shown, respectively. The parameters used are 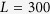 nm, 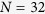, 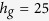 nm, and *f*_*g*_ = 0.5.

Figure 2(a,b) show the transmission spectra of the TE and TM waves. The black lines refer to the common DBR with a band gap between 860 nm and 940 nm. When the top surface grating is added, asymmetric line shapes appear (red lines), which can be explained in the Fano picture^2^,^22^. When the incident light is normal to the surface [see Fig. 1(b)], the grating-induced optical Bloch states at the 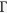 point (where the transverse wave vector is zero) are discrete. In the present structure, they appear in the DBR band gap and play the role of the discrete level in the Fano resonance. Hence, the asymmetric Fano line shapes [red lines in Fig. 2(a,b)] are the result of the coupling between the continuous transmission mode of the DBR and the discrete grating-induced Bloch levels at the 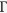 point. The effects of the Fano resonances depend on the transmissivity of the incident waves, being weaker in the DBR band gap owing to the low transmissivity. However, when another surface grating is added on the bottom of the DBR, almost symmetric resonant transmission peaks appear at the positions of the previous Fano resonances with peak transmissivities of 100% [blue lines in Fig. 2(a,b)]. We attribute this to the fact that the bottom grating also generates Fano resonances that are coherent with those generated by the top grating. These two Fano resonances interfere to form comb-like transmissions in the DBR band gap, that is, the transmission comb. When the incident light is not normal to the surface, the peaks in the transmission comb split, because the components of the wave vector along direction 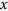 are different for *μ* = −1, 0, +1.

Because the comb-like shape is clearer in the DBR band gap, we will consider the transmission comb in the range from 860 nm to 940 nm. To this aim, we introduce the average full width at half maximum (FWHM), 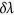, of the comb peaks and the average free spectral range, 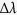, defined as the wavelength separation between adjacent transmission peaks. Figure 2(c,d) show 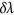 as a function of the grating height 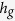 and duty cycle 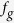. We can see that 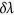 is different for the TE and TM waves due to the different TE and TM band structures of the DBR and is very small for both cases when 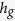 and 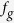 are small. Hence, the G/DBR/G structure provides a way to design multichannel optical filters with ultra narrow peaks. Considering the experimental conditions, we set *f*_*g*_ = 0.5 and 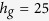 nm in the following calculations, where 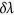 accounts to a few tenths of nanometer.

**Figure 3:**
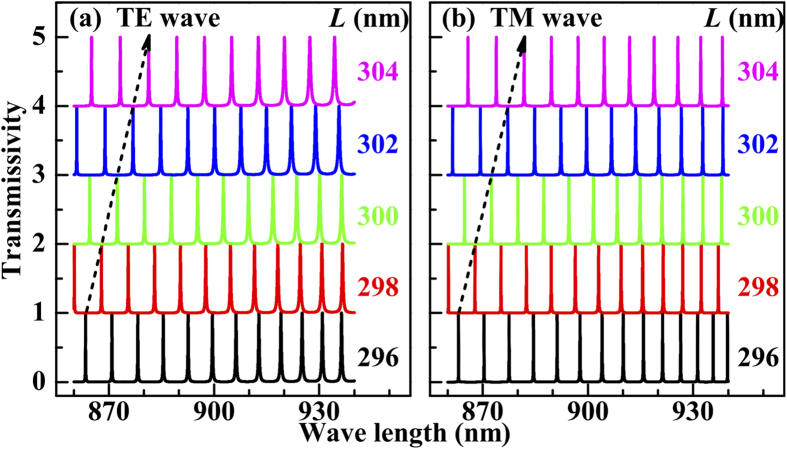
Transmission spectra of the (**a**) TE wave and (**b**) TM wave for several grating periods. For easy observation, the lines are offset in steps of 1. The dashed arrows in (**a**,**b**) show the shift direction for increasing grating period (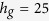 nm, *f*_*g*_ = 0.5, and 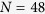).

The grating height and duty cycle do not affect the positions of the transmission peaks, which are determined by the grating period 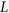. For both the TE and TM waves the transmission comb shifts towards the long wavelength side (red shift) with increasing 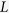, displayed by the dashed arrows in Fig. 3, since the horizontal wave vectors of the 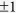 order modes (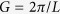) decrease. When the increment 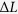 is far less than 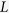, the redshifts are linear in 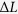 for both the TE and TM waves,


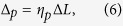


where 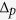 (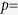 TE or TM) denotes the increment of the transmission comb position. The fitting parameter 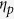 equals 2.25 and 2.34 for the TE and TM wave, respectively. Equation (6) indicates that the work region of the transmission comb can be controlled by adjusting the grating period.

**Figure 4:**
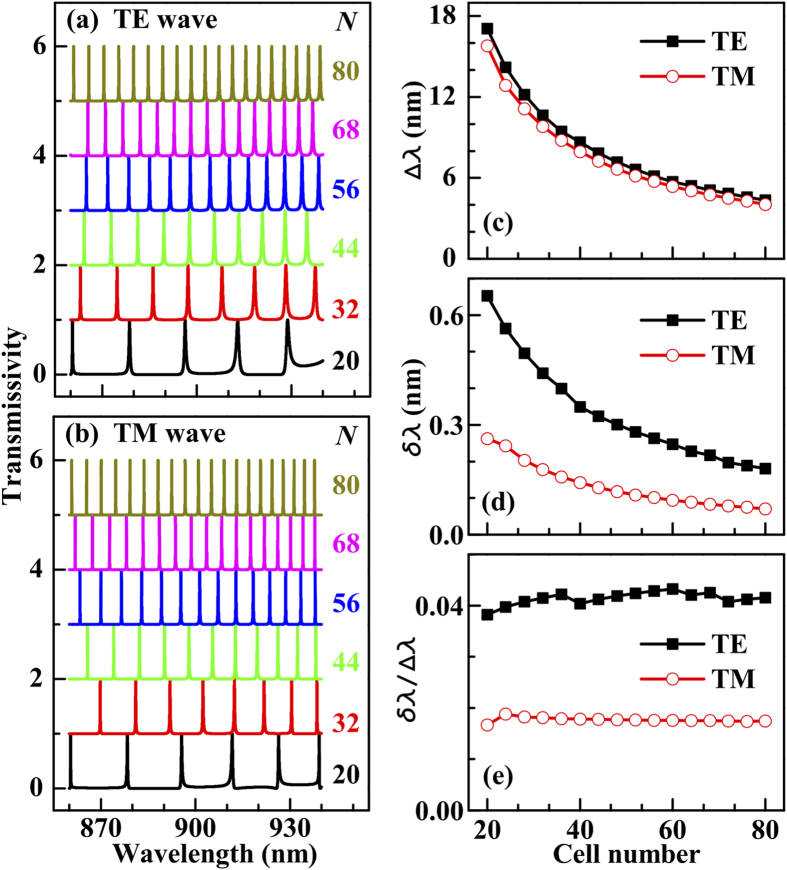
Transmission spectra of the (**a**) TE wave and (**b**) TM wave for several numbers of cells. In (**c**,**d**) the variations of the average free spectral range and FWHM are shown, respectively, and their ratio is plotted in (**e**). For easy observation, the lines in panels (**a**,**b**) are offset in steps of 1 (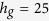 nm, *f*_*g*_ = 0.5, and 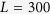 nm).

The transmission comb also strongly depends on the number of cells of the DBR, 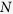. According to Fig. 4(a,b), the transmission peaks become denser and narrower when 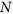 increases, corresponding to a decrease of 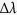 and 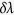. For the widely studied semiconductor microcavities^17^,^18^, 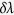 and 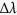 are mainly determined by the DBR reflectivity and the cavity thickness. Thus, it is impossible to decrease the two quantities simultaneously by increasing 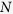. The DBR in the G/DBR/G structure fulfills the roles of the cavity layer and the two DBR mirrors in a semiconductor microcavity, with the help of the two surface gratings. Because the grating thickness (tens of nanometers) is small when the G/DBR/G and semiconductor microcavity have comparable 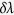 and 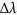, the height of the G/DBR/G is only about one third of that of the semiconductor microcavity^17^,^18^. For example, the total height of the G/DBR/G with 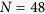 is 6.7 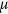
m, while that of the corresponding semiconductor microcavity is 20.8 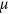
m.

Figure 4(c,d) show that 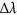 and 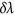 decrease with increasing 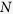. Since 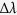 is almost the same for the TE and TM waves and measures the number density of the transmission peaks, the two transmission combs have a similar density, compare Fig. 4(a) with Fig. 4(b). However, the average FWHM of the TM spectrum is about half of that of the TE spectrum, both being less than 1 nm [see Fig. 4(d)] for the parameters (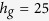 nm and 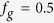) used in the calculation. The variation of the ratio 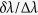 with 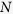 is plotted in Fig. 4(e). For the TE and TM waves, we obtain values of 0.04 and 0.02, respectively. Importantly, these two ratios hardly vary with 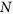, which indicates that it is practical to use the G/DBR/G to design a transmission comb with a certain peak number density by controlling the number of cells of the DBR, constituting a promising candidate for the ultra-narrow-band multichannel optical filters for both TE and TM waves.

**Figure 5:**
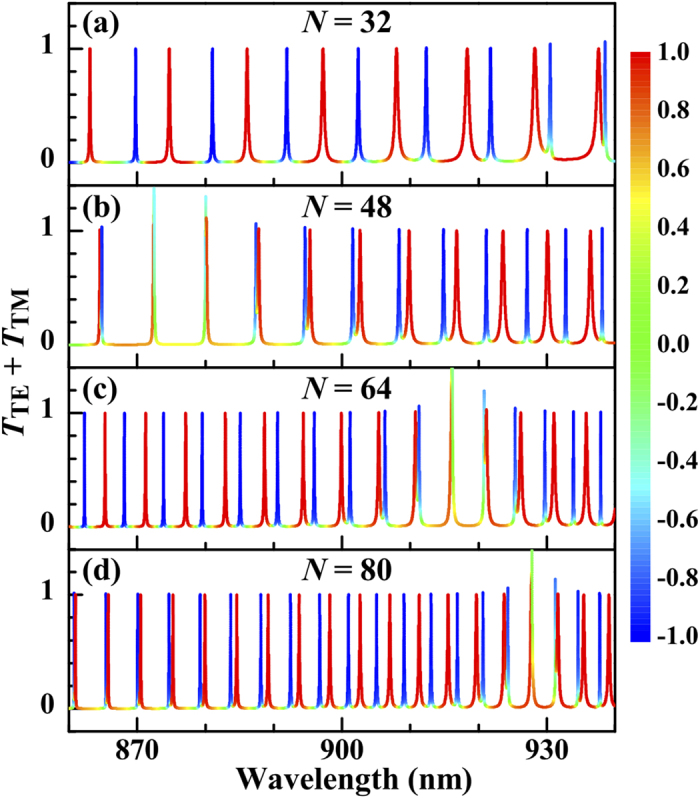
Variation of the sum *T*_TE_ + *T*_TM_ with the wavelength of the incident beam for different numbers of cells. The line colour represents the transmission polarization (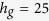 nm, *f*_*g*_ = 0.5, and 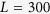 nm).

Comparing the transmission combs of the TE and TM waves shows that the transmission peaks do not coincide, which means that the G/DBR/G gives rise to a transmission polarization,


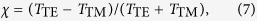


where 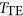 and 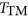 represent the transmissivities of the TE and TM waves, respectively. The polarization is represented by the line colour in Fig. 5 where we plot 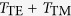. In certain regions the transmission peaks of the TE and TM waves are fully separated from each other, for examples in the wavebands 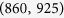 nm in Fig. 5(a), 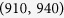 nm in Fig. 5(b), and 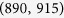 nm in Fig. 5(d). By Eq. (6), these polarized wavebands can be shifted by controlling the grating period and therefore the G/DBR/G can serve as a multichannel polarizer.

**Figure 6:**
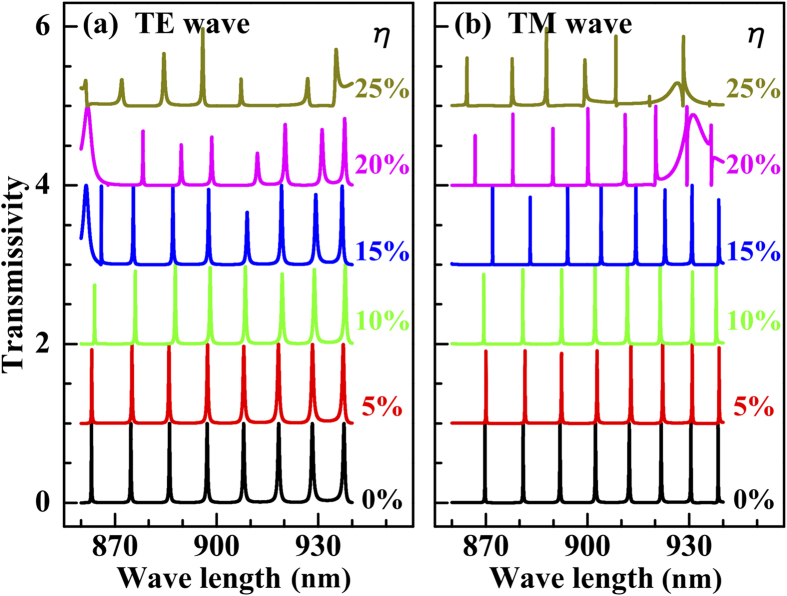
Transmission spectra of the (**a**) TE wave and (**b**) TM wave for randomness in the heights of the DBR layers, given by the parameter 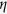. For easy observation, the lines are offset in steps of 1 (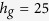 nm, *f*_*g*_ = 0.5, 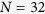, and 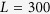 nm).

We next show that the transmission combs depend very weakly on randomness in the heights of the DBR layers. We define


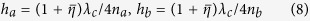


for the GaAs and AlAs layers, respectively, with 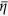 being a uniform random number in 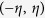. The results in Fig. 6 indicate that the transmission spectra maintain their comb-like forms at least up to 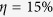. This weak dependence on the defects should make it possible to achieve the G/DBR/G experimentally. A relative shift between the positions of the top and bottom gratings will introduce an extra phase in the diffraction wave, which is small as long as the shift is small with respect to 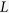.

## Discussion

By using two surface gratings we have achieved a transmission comb in the DBR band gap for TE and TM incident waves. The average FWHM of the transmission peaks in the comb is narrower than 1 nm and can go down to 0.1 nm. The total height of the proposed structure is only about one third of that of the widely used semiconductor microcavity for a similar comb-like transmission. The transmission of the proposed structure is fully polarized at the TE and TM transmission peaks. In addition, for both TE and TM waves the comb-like transmission is robust against randomness in the heights of the DBR layers (at least up to 15% randomness). As a result, the proposed structure is a promising candidate for ultra-narrow-band multichannel filters and polarizers.

## Additional Information

**How to cite this article**: Zhao, X. *et al.* Transmission comb of a distributed Bragg reflector with two surface dielectric gratings. *Sci. Rep.* **6**, 21125; doi: 10.1038/srep21125 (2016).

## References

[b1] Sheinfux H. H., Kaminer I., Plotnik Y., Bartal G. & Segev M. Subwavelength multilayer dielectrics: Ultrasensitive transmission and breakdown of effective-medium Theory. Phys. Rev. Lett. 113, 243901 (2014). 25541773 10.1103/PhysRevLett.113.243901

[b2] Dong G., Zhang Y., Kamran M. A. & Zou B. Tailoring of optical modes of semiconductor microcavities via metal and dielectric gratings. Opt. Lett. 37, 5085–5087 (2012). 23258013 10.1364/OL.37.005085

[b3] Kulishov M. & Kress B. Distributed Bragg reflector structures based on PT-symmetric coupling with lowest possible lasing threshold. Opt. Express 21, 22327–22337 (2013). 24104123 10.1364/OE.21.022327

[b4] O’Brien S. Comb transmission filters defined by phase-shifted superstructure Bragg gratings. Opt. Lett. 39, 1085–1088 (2014). 24562284 10.1364/OL.39.001085

[b5] Zhang H. *et al.* Efficient silicon nitride grating coupler with distributed Bragg reflectors. Opt. Express 22, 21800–21805 (2014). 25321555 10.1364/OE.22.021800

[b6] Armani D. K., Kippenberg T. J., Spillane S. M. & Vahala K. J. Ultra-high-Q toroid microcavity on a chip. Nature 421, 925–928 (2003). 12606995 10.1038/nature01371

[b7] Benson O. Assembly of hybrid photonic architectures from nanophotonic constituents. Nature 480, 193–199 (2011). 22158243 10.1038/nature10610

[b8] Chen D. & Han J. High reflectance membrane-based distributed Bragg reflectors for GaN photonics. Appl. Phys. Lett. 101, 221104 (2012).

[b9] Sheng X., Johnson S. G., Broderick L. Z., Michel J. & Kimerling L. C. Integrated photonic structures for light trapping in thin-film Si solar cells. Appl. Phys. Lett. 100, 111110 (2012).

[b10] Antón C. *et al.* Dynamics of a polariton condensate transistor switch. Appl. Phys. Lett. 101, 261116 (2012).

[b11] Steger M. *et al.* Single-wavelength, all-optical switching based on exciton-polaritons. Appl. Phys. Lett. 101, 131104 (2012).

[b12] Zheng Y., Kawashima T., Satoh N. & Kan H. Stable-wavelength and narrow-bandwidth high-power external-cavity laser diode stack. Appl. Phys. Express 7, 092702 (2014).

[b13] Chen L. & Towe E. Nanowire lasers with distributed-Bragg-reflector mirrors. Appl. Phys. Lett. 89, 053125 (2006).

[b14] Gessler J. *et al.* Low dimensional GaAs/air vertical microcavity lasers. Appl. Phys. Lett. 104, 081113 (2014).

[b15] Boonruang S. & Mohammed W. S. Multiwavelength guided mode resonance sensor array Appl. Phys. Express 8, 092004 (2015).

[b16] Levy-Yurista G. & Friesem A. A. Very narrow spectral filters with multilayered grating-waveguide structures. Appl. Phys. Lett. 77, 1596 (2000).

[b17] Lai C. W. *et al.* Coherent zero-state and *π*-state in an exciton-polariton condensate array. Nature 450, 529–532 (2007). 18033292 10.1038/nature06334

[b18] Deng H., Haug H. & Yamamoto Y. Exciton-polariton Bose-Einstein condensation. Rev. Mod. Phys. 82, 1489 (2010).

[b19] Reichert J. *et al.* Phase coherent vacuum-ultraviolet to radio frequency comparison with a mode-locked laser. Phys. Rev. Lett. 84, 3232–3235 (2000). 11019058 10.1103/PhysRevLett.84.3232

[b20] Thorpe M. J., Moll K. D., Jones J. J., Safdi B. & Ye J. Broadband cavity ringdown spectroscopy for sensitive and rapid molecular detection. Science 311, 1595–1599 (2006). 16543457 10.1126/science.1123921

[b21] Diddams S. A., Hollberg L. & Mbele V. Molecular fingerprinting with the resolved modes of a femtosecond laser frequency comb. Nature 445, 627–630 (2007). 17287805 10.1038/nature05524

[b22] Nicolas R. *et al.* Plasmonic mode interferences and Fano resonances in metal-insulator-metal nanostructured interface. Sci. Rep. 5, 14419 (2015). 26399425 10.1038/srep14419 PMC4585844

[b23] Pruessner M. W., Stievater T. H. & Rabinovich W. S. Integrated waveguide Fabry-Perot microcavities with silicon/air Bragg mirrors. Opt. Lett. 32, 533–535 (2007). 17392912 10.1364/ol.32.000533

[b24] Zhang X., Liu H., Tian J., Song Y. & Wang L. Band-selective optical polarizer based on gold-nanowire plasmonic diffraction gratings. Nano Lett. 8, 2653 (2008). 18700802 10.1021/nl0808435

[b25] Handbook of optical constants of solids, edited by Palik E. D. (Academic, New York, 1985).

